# Cardiovascular disease in diffuse idiopathic skeletal hyperostosis (DISH): from theory to reality—a 10-year follow-up study

**DOI:** 10.1186/s13075-020-02278-w

**Published:** 2020-08-17

**Authors:** Karina Glick, Irina Novofastovski, Naama Schwartz, Reuven Mader

**Affiliations:** 1 Department of Medicine A, Assuta University Hospital, Ben-Gurion University of the Negev Faculty of Health Sciences, Ashdod, Israel; 2 Rheumatic Diseases Unit, Ha’Emek MC, 18101 Afula, Israel; 3 Ha’Emek MC, 18101 Afula, Israel

**Keywords:** Diffuse idiopathic skeletal hyperostosis, Cardiovascular disease, Ischemic heart disease, Myocardial infarction, Atherosclerosis, Metabolic syndrome

## Abstract

**Objective:**

To describe actual cardiovascular events over a decade in patients with diffuse idiopathic skeletal hyperostosis (DISH), without previously known CV diseases.

**Methods:**

The medical records of patients with DISH and controls, beginning in 2006 (without known CV disease), were reviewed. Demographic, constitutional, and laboratory data were collected. Comparison of CV events following 2006 was performed according to the outcome definitions set by the Framingham score 2: coronary event demonstrated by a coronary imaging modality, acute myocardial infarction (MI), coronary death, congestive heart failure with a reduced ejection fraction, and angina pectoris.

**Results:**

Data were available for 45 patients with DISH and 47 controls without DISH from the original cohort (91.8% and 97.9% respectively). By the Framingham score, 28.6% (± 20.33) of the DISH patients were expected to be affected with CVD at 10 years of follow-up. We observed that nearly 39% of them developed CVD during that period (95% CI 23.8–53.5%). The incidence of MI over the 10-year period was significantly higher in the DISH group (*P* = 0.005). The DISH group had higher morbidity with a higher composite outcome of 38.8% vs 25.5% in the control cohort, and the number of non-elective hospital admissions per patient, despite neither reaching statistical significance.

**Conclusion:**

Our study showed that the Framingham score underestimates the real risk for developing CVD in patients with DISH, specifically the risk for MI. We propose more scrutiny is warranted in evaluating CV risk in these patients, more demanding treatment target goals should be established, and earlier and more aggressive medical interventions should be undertaken, particularly primary prevention. Larger prospective studies are needed to corroborate these findings.

## Key points

- This is the first study that compares the probability for developing CV disease with the actual CV disease events in patients with DISH.
- Patients with diffuse idiopathic skeletal hyperostosis (DISH) have a significantly higher risk to develop myocardial infarction.
- Myocardial infarction was higher than expected based on traditional risk stratification.
- DISH should be evaluated as an independent risk factor for myocardial infarction.

## Introduction

DISH is characterized by ossification and calcification of the entheses. It is more common in males, its prevalence increasing with age, with an average of 10% above 70 years of age [1]. Although DISH has been described as an axial disease, it has several extra-axial musculoskeletal manifestations such as peripheral enthesopathies, involvement of joints not usually affected by osteoarthritis, and hypertrophic joints’ changes [2]. Furthermore, DISH has been reported to be associated with obesity and type 2 diabetes mellitus (DM) [3–5], in particular in patients with central obesity. Several factors that might promote osteogenesis have been reported, including hyperinsulinemia. It has also been shown that the prevalence of hypertension and hyperlipidemia is higher in patients with DISH [6–9]. These factors, obesity, hypertension (HTN), and DM, are the main components of the metabolic syndrome (MS) [10]. The Framingham risk score is used to evaluate the 10-year risk for developing cardiovascular disease (CVD) [11]. In fact, this score was found to be more reliable in predicting CVD than MS [12]. A case-control study we conducted on patients with DISH without CVD demonstrated a significantly higher prevalence of metabolic syndrome and a significantly higher Framingham score compared with the control group [9]. The purpose of the present study is to describe the real incidence of CVD during 10 years of observation.

## Materials and methods

The study was approved by the local Institutional Review Board (approval numbers, 3960705; 012316-EMC). A written informed consent was obtained from all participants in the original cohort.

The primary outcome measure was the incidence at 10 years of CV events in the DISH group compared with the non-DISH group. Secondary outcomes were mortality, newly diagnosed DM, number of non-elective hospital admissions, and changes in medical treatments.

The 2006 cohort has already been described [9]. Briefly, all the patients with DISH fulfilled the Resnick classification criteria. The control group was composed of patients with osteoarthritis without DISH. The main inclusion criterion was the absence of CVD in all the participants. The main outcome measures were the presence of MS by two methods and the cardiac heart disease risk by the Framingham score.

The electronic medical records of all the patients in the original cohort were reviewed in 2016. The electronic medical records of the available patients were accessible from 2006 to the date of the data collection. We reviewed diagnoses, admission and discharge notes, community and outpatients’ office visits, laboratory data, and medications prescribed and dispensed from the pharmacies. Data were available for 45 patients with DISH and 47 controls without DISH from the original 2006 cohort (91.8% and 97.9% respectively). Cardiovascular events were collected and stratified according to the outcome definitions set by the Framingham risk score (FRS) for cardiovascular disease [11]: coronary event demonstrated by a coronary angiography or another coronary imaging modality, acute myocardial infarction (MI), coronary death, congestive heart failure with a reduced ejection fraction (HFrEF), and angina pectoris. These outcomes had to be diagnosed by either a cardiologist or an internist. Cerebral vascular event (CVA), transient ischemic attack (TIA), and hemorrhagic stroke were included if diagnosed by either a neurologist or an internist. Also, peripheral vascular disease (PVD), diagnosed by an internist or a vascular surgeon, was included. It bears mentioning that in the historical study, the FRS (for both groups) was calculated according to the accepted parameters published in 1998. We re-calculated it using data collected in 2006 according to the more validated and accepted score of 2008 [11]. While the 1998 FRS was designed to identify patients at risk for coronary heart disease, the 2008 score predicts the risk for a cardiovascular event and not for coronary heart disease only.

Non-elective hospital admissions were reviewed for neurology, cardiology, internal medicine, and vascular surgery. A composite outcome was calculated, as in the Framingham study 2, and included MI, CVA, TIA, PVD, and HFrEF. The statistical analysis of the Framingham risk score was performed using C statistics [13]. The rest of the demographic data, medication use, and laboratory parameters were collected.

Categorical variables were presented by frequency and percentages and analyzed by the chi-square test (or Fishers’ exact test). Continuous variables were presented as mean, standard deviation, and median and analyzed by the Student *T* test or Wilcoxon two-sample test. In order to adjust for the effect of other risk factors, more common in the DISH group, a multiple logistic regression analysis that included obesity, hypertension, and DM was performed. The Framingham score accuracy was also validated by utilizing the area under the receiver operating characteristic (ROC). Statistical significance was set at *P* < 0.05. The statistical analysis was performed with SAS 9.4 software (SAS Institute Inc. Cary, NC, USA).

## Results

**Fig. 1:**
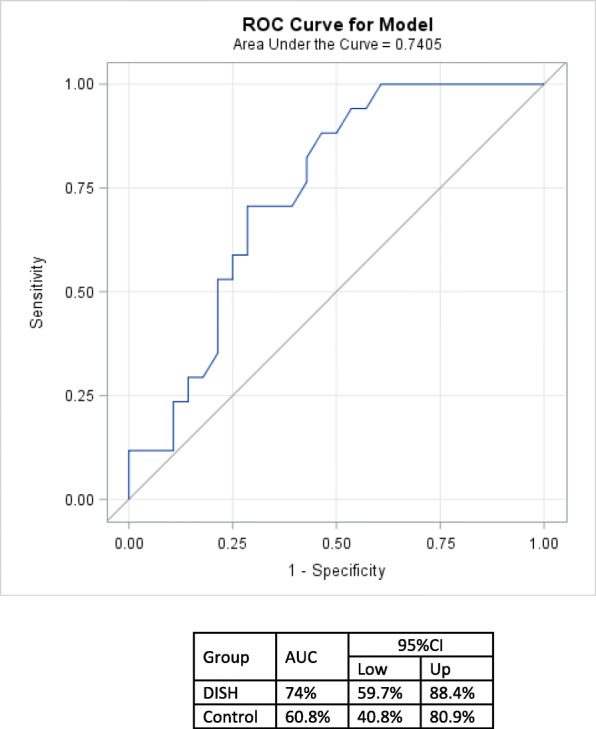
Composite outcome prediction of the Framingham risk score

According to the Framingham score, 28.6% of the 2006 DISH patients were expected to be affected with CVD at 10 years of follow-up (Table 1). In reality, nearly 39% of them developed CVD, with the area under the (ROC) curve reflecting a fair 74% accuracy (Fig. 1). This means an underestimation of the risk. Logistic regression showed that for every 1% increase in the CV risk calculated by the Framingham score, the odds of CVD in the DISH group are 1.04 (95% CI 1.005–1.08; *P* = 0.0244) times higher. The groups did not differ in age, gender distribution, and serum lipid profile. In both the historic and the present cohorts, HbA1c and BMI were significantly higher in the DISH group. The 2008 re-calculated FRS of the original cohort was significantly higher in DISH patients compared with non-DISH patients.

**Table 1:** Characteristics of the DISH group and control

		DISH *N* = 45 (mean, SD, median)	Control *N* = 47 (mean, SD, median)	*P* value
	Age at 2006	64.02 (9.76, 63)	62.13 (8.6, 60)	NS
	Age at 2016	73.87 (9.21, 73)	72.21 (8.08, 71)	NS
	Men (%)	13 (28.9%)	11 (21.3%)	NS
	BMI at 2006	34.02 (6.24, 32)	30.74 (5.02, 30)	0.0069
	Smokers 2006	13 (28.9%)	16 (34.0%)	NS
	FRS calculated at 2006	28.67 (20.33, 23)	17.85 (16.31, 12)	0.0028
HTN	Systolic blood pressure 2006 mmHg	141.51 (16.49, 141)	132.09 (20.43, 129)	0.0171
	Diastolic blood pressure 2006 mmHg	82.06 (9.83, 82)	80.13 (10.47, 78)	0.0576
Total blood cholesterol	2006 (mg/dl) (mean, SD)	214.38 (38.98, 211)	208.28 (35.95, 206)	NS
	2016 (mg/dl) (mean, SD)	174.33 (40.1, 169)	190.34 (38.32, 185)	NS
HDL levels	2006 (mg/dl) (mean, SD)	51.2 (12.19, 48)	51.81 (13.47, 49)	NS
	2016 (mg/dl) (mean, SD)	47.51 (14.52, 44)	51.26 (12.7, 48)	NS
LDL levels	2006 (mg/dl) (mean, SD)	128.07 (33.78, 132)	126.34 (30.21, 129)	NS
	2016 (mg/dl) (mean, SD)	102.88 (30.87, 101)	111.66 (34.59, 113)	NS
HbA1c levels	2006 mg% (mean, SD)	6.35 (0.98, 6)	5.73 (0.44, 5.7)	0.0004
	2016 mg% (mean, SD)	6.51 (0.99, 6.3)	5.9 (1.16, 5.7)	0.0005

*BMI* body mass index, *FRS* Framingham risk score, according to the overall CVD risk [11], *HTN* hypertension, *HDL* high-density lipoprotein, *LDL* low-density lipoprotein, *HbA1c* glycated hemoglobin

Serum CRP levels were available in 42 DISH patients and 46 in non-DISH patients and were higher in DISH vs non-DISH patients (6 ± 9.6 vs 4.4 ± 4.4 mg/l respectively). Based on the Wilcoxon two-sample test, there was no significant difference in the CRP between the two study groups (*P* = 0.6608).

A comparison between the groups showed that over a 10-year period, 11 new cases of DM emerged in the DISH group compared with 4 in the non-DISH group (*P* < 0.01). The number of patients who developed HTN during the follow-up period did not differ between the groups; however, HTN was more prevalent in the DISH group as compared with the control group (*P* ≤ 0.05). The incidence of patients with dyslipidemia or HFrEF did not differ between the groups, but significantly more patients were treated with renin-angiotensin system inhibitors in the DISH group (Table 2).

**Table 2:** Concomitant diagnoses and medications at 2006 and during follow-up

		DISH *N* = 45 (%)	Control *N* = 47 (%)	*P* value
Dyslipidemia	2006 *N* (%)	(48.9) 22	(29.8) 14	0.06
	2016 N (%)	(81.1) 36	(69.6) 32	0.176
HTN	2006 *N* (%)	(62.2) 28	(38.3) 18	0.02
	2016 *N* (%)	(86.7) 39	(59.6) 28	0.003
Type 2 DM	2006 *N* (%)	(33.3) 15	(8.5) 4	0.003
	2016 *N* (%)	(59.1) 26	(17) 8	< 0.0001
Heart failure (HFrEF + HFpEF)	2006 *N* (%)	0 (0)	0 (0)	
	2016 *N* (%)	13 (28.9)	12 (25.5)	0.717
Aspirin treatment	2006 *N* (%)	(35.6) 16	(19.2) 9	0.077
	2016 *N* (%)	(60) 27	(44.7) 21	0.598
ACEi/ARB treatment	2006 *N* (%)	(31.1) 14	(14.9) 7	0.063
	2016 *N* (%)	(62.2) 28	(27.7) 13	0.0009
HMG-CoA reductase inhibitor treatment	2006 *N* (%)	(40) 18	(25.5) 12	0.138
	2016 *N* (%)	(75) 33	(51.1) 24	0.0185
Metformin treatment	2006 *N* (%)	10 (22.2)	3 (6.4)	0.029
	2016 *N* (%)	12 (26.7)	4 (8.5)	0.021

*HFrEF* heart failure with reduced ejection fraction, *HFpEF* heart failure with preserved ejection fraction, *ACEi/ARB* angiotensin-converting enzyme inhibitor/angiotensin receptor blocker

The cardiovascular outcomes are depicted in Table 3. It clearly demonstrates that the incidence of MI and the composite outcome over the 10-year period was significantly higher in the DISH group (*P* < 0.01). The secondary outcomes did not differ between the groups. The higher morbidity of the DISH group was evident in the composite outcome (38.8% vs 25.5%), as well as the number of non-elective hospital admissions per patient, though neither reached statistical significance. As shown in Table 3, the sole outcome with significant association to DISH was MI. Therefore, a multiple logistic regression model was implemented, including DISH, obesity, type II diabetes, and dyslipidemia as predictors. The DISH predictor was borderline significant (*P* = 0.0425), whereas the rest of the potential predictors were not significant (obesity *P* = 0.9601, type II diabetes *P* = 0.7501, and dyslipidemia *P* = 0.2957). After adjusting to obesity, type II diabetes, and dyslipidemia, for patients with DISH, the odds of having MI was 6.03 (95% CI 1.06–34.2) times higher compared to the control group (Hosmer and Lemeshow goodness-of-fit test *P* value = 0.8288; C statistic = 76%).

**Table 3:** Cardiovascular outcomes during the 10-year follow-up

	DISH *N* = 45 *N* (%)	Control *N* = 47 *N* (%)	*P* value
Myocardial infarction	11 (24.4)	2 (4.3)	0.0055
Transient ischemic attack	5 (11.1)	9 (19.2)	0.2833
Cerebrovascular accident	4 (8.9)	2 (4.3)	0.4297
Peripheral vascular disease	1 (2.2)	0 (0)	0.4891
Heart failure with reduced ejection fraction	3 (6.7)	0	0.1130
All-cause mortality	5 (11.1)	4 (8.5%)	0.7369
CV mortality	3 (6.7)	0 (0%)	0.1130
Composite outcomes*	17 (38.8)	12 (25.5)	0.2063

*A subject with an outcome was counted once, even if had more than one outcome during the follow-up. *P* value calculated by the chi-square test (or Fisher’s exact test—depending on the percent of cells with an expected count less than 5)

## Discussion

In 2006, we collected data on a cohort of patients with DISH. In our original cohort, patients with DISH were more often affected by metabolic syndrome, by either the National Cholesterol Education Program III definition or the World Health Organization definition, compared with non-DISH patients (*P* = 0.001 and *P* = 0.007 respectively). As a result, the Framingham score in patients with DISH vs non-DISH patients conferred a significantly higher coronary heart disease (CHD) risk (*P* = 0.007). Using the more updated version of the Framingham score, we showed a higher risk for cardiovascular disease (not only CHD risk) in patients with DISH compared with age- and sex-matched non-DISH patients. In fact, the Framingham score in the 2006 cohort underestimated the extent of CVD at 10 years. The Framingham score has been recently validated in Israeli males [14]. The association of DISH with obesity, DM, HTN, and dyslipidemia is well described [6, 15, 16]. Thus, there is a greater probability of CVD risk in these patients than in the general population. Furthermore, the incidence of CV events was even higher than expected in this population. Cardiac events were significantly more frequent, but no other organ system involvement. A previous cross-sectional study showed that in patients recruited after either coronary bypass surgery, cardiac valve replacement, or with CHF, a higher than expected prevalence of DISH was reported in nearly a third of them [17]. A higher than expected prevalence of DISH has also been reported in patients with thoracic aortic aneurysm, compared to patients without aortic aneurysm [18]. In contrast, another study could not find support for an association between DISH and abdominal aorta atherosclerosis.

Patients with DISH did have an increased prevalence of DM when recruited to the study. It is noteworthy that in the follow-up period, the likelihood of patients with DISH of developing new DM was high. These data reiterated previous reports that support the association between DM and DISH, suggesting that DISH might be considered a pre-diabetic state.

It has already been shown that an inflammatory state can accelerate atherosclerosis [19–22]. It has recently been suggested, by ultrasonography [23] and MRI [24] studies in DISH, that entheseal inflammation may precede the ossification process and some similarities with spondyloarthropathies have been found [25–27]. Therefore, another possible explanation for the higher rate of CVD in DISH, beyond the traditional risk factors for CVD, is persistent low-grade inflammation that may exist in patients with DISH. This contradicts the current belief that DISH is a non-inflammatory condition.

Elevated CRP levels are considered to be associated with increased CV risk. Higher CRP levels are associated with a higher BMI and more so in female patients [28]. It is therefore not surprising that the CRP levels in the DISH group, with a higher BMI, had higher CRP levels, though they did not reach statistical significance. Coronary artery calcifications are considered to be a strong risk factor for adverse cardiovascular outcomes. It has recently been shown that patients with DISH have a significantly higher risk of having coronary artery calcifications, even after correction for age, gender, and atherosclerotic risk factors [29]. Thus, DISH itself may contribute to the CVD risk burden.

To the best of our knowledge, our study is the first to describe actual CV events, over 10 year’s period, in patients with DISH without previously known CVD. The 2 groups had a similar age and gender distribution. It can be argued that a higher coronary artery disease incidence was expected, based on the higher prevalence of CV risk factors in the DISH group. The fact that the real occurrence of CHD was higher than predicted suggests that DISH may be an independent risk factor.

The study has some limitations. First, the collection of the data was retrospective. We believe that the meticulous definition of the outcome measures and the quality of the documentation prevented a significant distortion of results. Second, our cohort has a preponderance of female patients, which is in contrast to the usual gender distribution in DISH. Nevertheless, the prevalence of CVD in the cohort’s age group is less gender dependent, and the groups were comparable for age and gender which mitigated the potential deviation of the results.

## Conclusions

The study showed that the Framingham score underestimates the real risk for developing CVD in patients with DISH, in particular for myocardial infarction. We propose more scrutiny is warranted in evaluating CV risk in these patients, more demanding treatment target goals should be established, and as a result, earlier and more aggressive preventive medical interventions instituted. Larger, prospective longitudinal studies are needed to strengthen these findings.

## Data Availability

Data will not be shared because it belongs to a data base containing more details for future analysis.

## Abbreviations

CHD: Coronary heart disease
CHF: Congestive heart failure
CV: Cardiovascular
CVD: Cardiovascular disease
CVA: Cerebral vascular event
DISH: Diffuse idiopathic skeletal hyperostosis
DM: Diabetes mellitus
MS: Metabolic syndrome
HTS: Hypertension
HFrEF: Congestive heart failure with reduced ejection fracture
MI: Myocardial infarction
TIA: Transient ischemic attack
PVD: Peripheral vascular disease
